# Biomechanical estimation of tennis serve using inertial sensors: A case study

**DOI:** 10.3389/fspor.2022.962941

**Published:** 2022-12-02

**Authors:** Franck Brocherie, Daniel Dinu

**Affiliations:** Laboratory Sport, Expertise and Performance (EA 7370), French Institute of Sport, Paris, France

**Keywords:** inertial navigation system, 3D kinematic analysis, posture, accuracy, center of mass, biomechanics

## Abstract

Inertial measurement units may provide a relevant on-court 3-Dimension measurement system for tennis serve biomechanical analysis. Therefore, this case study aimed to report the feasibility of inertial measurement unit's kinematic and kinetic data collection during tennis serve. Two injury-free highly-trained tennis players were equipped with the inertial measurement unit (Xsens MVN suit) and performed 2 trials of five flat “first” serves on a 1 m^2^ target zone bordering the service box of an indoor GreenSet^®^ tennis court surface. With the exception of the center of gravity rotation at the loading stage, all joint (shoulder, elbow, knee) angles, center of mass displacements and rotations followed a similar development for both female and male participants from loading to finish stages. At ball contact stage, articular moments (mid-trunk, upper-trunk, shoulder, elbow, wrist) and segmental contribution (pelvis linear, pelvis rotation, trunk, shoulder, elbow, wrist) repartitions also showed a comparable movement. From loading to finish stages, total, lower and upper energy contribution were similar for both players, with coefficient of variations deemed acceptable between the two trials. This inertial measurement unit appears suitable for on-court tennis serve biomechanical data collection and subsequent analysis to provide tennis players and practitioners tailored feedbacks to facilitate motor learning process and develop serve efficiency.

## Introduction

With over 50 million practitioners over the word, tennis is the most popular individual sport (1). Although physical fitness has a preponderant role in tennis success, technical proficiency remains the predominant factor (2). In this view, the serve is a key element of match success (3) that gives the opportunity during each point to directly win it or to markedly influence the subsequent strokes. Toward maintaining high levels of power and serve velocity without sacrificing accuracy, serve biomechanics play an integral role. While comprehensive and detailed reviews of tennis biomechanics have been previously reported [e.g., (3, 4)], and because injuries have most often a “pathomechanical” cause (5), a better understanding of tennis serve biomechanics in the most ecological setting is still necessary in regard to player development and injury prevention.

To date, the tennis serve's “*kinetic chain*” has been investigated through laboratory-bound marker-based motion capture, sometimes associated with force plate measurements, raising some ecological validity concerns. Most tennis serve-related studies have focused on discrete body [e.g., ankle, shoulder, elbow; (6)] and ball and racquet kinematics (i.e., positions, durations, accelerations, velocities) (7). However, in order to maximize serve's power, the “*leg drive*”, the upper arm and the trunk rotations during the tennis serve are subtle mechanical coaching points (2) which need to be appraised. Briefly, a forceful “*leg drive*” (8) and frontal/sagittal plane trunk rotations (9) are critical for transferring the angular momentum from the legs to the upper limbs, participating in greater racket and ball velocities at impact (10). Any improper kinematic patterns susceptible to increase joint kinetics (i.e., rotations, forces, moments, energies) without improving ball velocity could be viewed as counterproductive and possibly “pathomechanical” (11). In recent years, wearable inertial measurement units (IMUs; embedding 3D accelerometers, gyroscopes and eventually magnetometers) allow one to estimate segment orientation and joint angular kinematics by exploiting the laws governing the motion of a rotating rigid body (12, 13). In this view, IMUs and magnetic measurement units have gained popularity for human motion capture solution in clinical practice (14) and sports settings (15) such as shot put (16) or basketball shooting (17). Although several validation or reliability studies have been conducted [e.g., (16, 18)], sport-specific demands require further data to support their use in practical settings. For instance, the Xsens suit (MVN Biomech) may provide a relevant on-court, 3D measurement system for tennis serve analysis.

Studies having investigated tennis serve using IMUs are sparse (19, 20), but demonstrated some kinematic synergies implications for the practitioners. However, none of these studies used whole-body IMUs for an entire quantification of the “*kinetic chain*” during the tennis serve. Because we envision that such easy-to-use wearables IMUs would permit to achieve a better understanding of the tennis serve for the development of stroke production with minimal risk of injury, the purpose of this case study was to report the feasibility to quantify IMUs whole-body kinematic and kinetic data during on-court tennis serve. Reporting its effectiveness may offer useful ecological individualized biomechanical and technical information/instruction to tennis practitioners in the tennis serve's motor learning process.

## Methods

### Participants

Two injury-free right-handed tennis players (1 male, 18 years, 177 cm, 65 kg; and 1 female, 17 years; 173 cm, 60 kg) with extensive regular training and competition participated in the study. A tennis level at least equivalent to the International Tennis Federation Association (ITF) category level 3–4 (“2^è*me*^ série” in the French Tennis Federation classification) was requested in order to guarantee enough accuracy in stroke production and precision, as well as to be representative of highly-trained tennis standards. The two participants were informed about the aim of the study and the protocol after which they or parent/guardian provided a written informed consent. The study was approved by the Local Ethical Committee and conducted in accordance with the Declaration of Helsinki.

### Experimental procedure

After a standardized 15 min warm-up (including mobility exercises, tennis-specific drills and progressive serves), participants were equipped with the IMU, and then requested to perform two trials of five flat “first” serves movements (interspersed with 10 s in-between for replacement and recovery) using a foot-back technique on a 1 m^2^ target zone bordering the T of the “deuce” service box of an indoor GreenSet^®^ tennis court surface. To ensure standardized playing conditions, temperature (24°C), relative humidity (44%) and light (>500 lux) were controlled and new balls (US open^®^) were used.

### Instrumentation

Participants were required to wear a MVN Biomech Link wireless data link suit (Xsens Technologies BV, Enschede, The Netherlands). This suit is composed of 17 miniature IMUs, a transmission pack and battery zipped on a compression suit (21). Each IMU contains three gyroscopes, three accelerometers and three magnetometers in a 35 g box about the size of a matchbox. Each IMU captures the 6-degrees-of-freedom of the body segment to which it is fixed, in real time at a sampling frequency of 240 Hz. Based on Zatsiorsky–Seluyanov's inertial parameters reported by de Leva (22), biomechanical calibration procedure (i.e., sensor to body alignment and body dimension determination) was done from a T-pose (i.e., upright with arms horizontally and thumbs forward) or N-pose (i.e., arms neutral besides body) followed by specific movements as described elsewhere (23). For each participant, trial and serve movement, motion data was recorded for the entire serve cycle (i.e., from preparation to follow-through stages with specific capture on loading, ball contact and finish stages) (24).

Racquet velocity at ball contact was recorded for each trial using a radar (Stalker ATS II, Applied Concepts, Dallas, TX, USA accuracy: ± 1 mph, frequency: 34.7 GHz, Target Acquisition Time: 0.01 s) attached to a 2.5-m height tripod, 2 m behind the serve line in the direction of the serve. The fastest successful tennis serve movement (i.e., having reached the target zone) of each trial was retained for subsequent analysis.

### Determination of variables

After extraction of the tennis serve-related motion data with MVN Biomech software, a customized MatLab™ (version R2010A Natick, Massachussetts, USA) program was used to calculate the knees (both for serve and opposite sides), shoulders and elbow joint angles using a Newton-Euler method (21). Furthermore, articular forces and moments were calculated for wrists, elbows, shoulders, mid-trunk and upper-trunk. Specifically, knowing the kinematics and inertial properties of the segments of the biomechanical model, forces and moments were derived based on the Newton-Euler equations:


(1) $$\sum F_{i}=m_{i}a_{i}$$

(2) $$\begin{array}{l} F_{i+1}+m_{i}g+F_{i}=m_{i}a_{i} \end{array}$$

where *F*_*i*_ corresponds to the striking force in the case of the first segment and the force taken up by the distal joint for the other segments, *F*_*i*+1_ corresponds to the force taken up by the proximal joint, *m*_*i*_ the mass of the segment studied and *a*_*i*_ the acceleration of the segment studied at its center of gravity.

Segmental contributions were also determined for pelvis linear and rotation, trunk, shoulder, elbow and wrist. Specifically, segmental contribution was computed by the analytical calculation of the velocity of each segment of interest settings (15):


(3) $$\begin{array}{l} {\vec{V}}_{seg\;x}={\vec{V}}_{pelvis}+{\vec{\omega }}_{pelvis}\times {\vec{L}}_{pelvis}+{\vec{\omega }}_{seg\;1}\times {\vec{L}}_{seg\;1}\\ \;+\ldots +{\vec{\omega }}_{seg\;x-1}\times {\vec{L}}_{seg\;x-1} \end{array}$$

where $\vec{V}$ represents the linear velocity, $\vec{\omega }$ represents the 3D segment angular velocity, and $\vec{L}$ represents the length corresponding to the 3D vector between ${\vec{V}}_{seg\;x}$ and the proximal joint.

Then, a kinematic chain was built for each segment with the pelvis used as reference point. The contribution of each segment was found by projecting the velocity vector of the segment on the velocity vector of the wrist. For example, the projected velocity of the upper arm has been calculated as follows:


(4) $$\begin{array}{l} {\vec{V}}_{upper\;arm\;proj.}=\frac{{\vec{V}}_{upper\;arm}{\times \vec{V}}_{hand}}{||{\vec{V}}_{hand}|{|}^{2}}\times {\vec{V}}_{hand} \end{array}$$

Where ${\vec{V}}_{upper\;arm}$ and ${\vec{V}}_{hand}$ are the velocity vectors of the upper arm and the hand, respectively, both determined by multiplying 3D segment angular velocity of the segment of interest.

Total kinetic energy (*T*_*i*_) of each segment was calculated as the sum of the kinetic energy due to the rotation of the segment and the kinetic energy due to its translation:


(5) $$\begin{array}{l} T_{i}=\;\frac{1}{2}\vec{\omega }\;{\vec{H}}_{G,i}+\;\frac{1}{2}{\vec{V}}_{Gi}\;{\vec{G}}_{i} \end{array}$$

The kinetic energy of the whole-body is then given by the sum of each segment's *T*_*i*_.

### Statistical analysis

Absolute and relative (percentage differences between trials 1 and 2) values of articular moments, segmental contributions and energetic transfer, as well as mean and standard deviation (SD) are presented by descriptive statistics. Coefficient of variation (CVs) (25) were calculated as (CV = SD/mean) and interpreted as follows: 0–4.9%, “*excellent*”; 5.0–9.9%, “*very good*”; 10.0–14.9%, “*good*”; 15.0–24.9%, “*acceptable*”.

## Results

**Figure 1:**
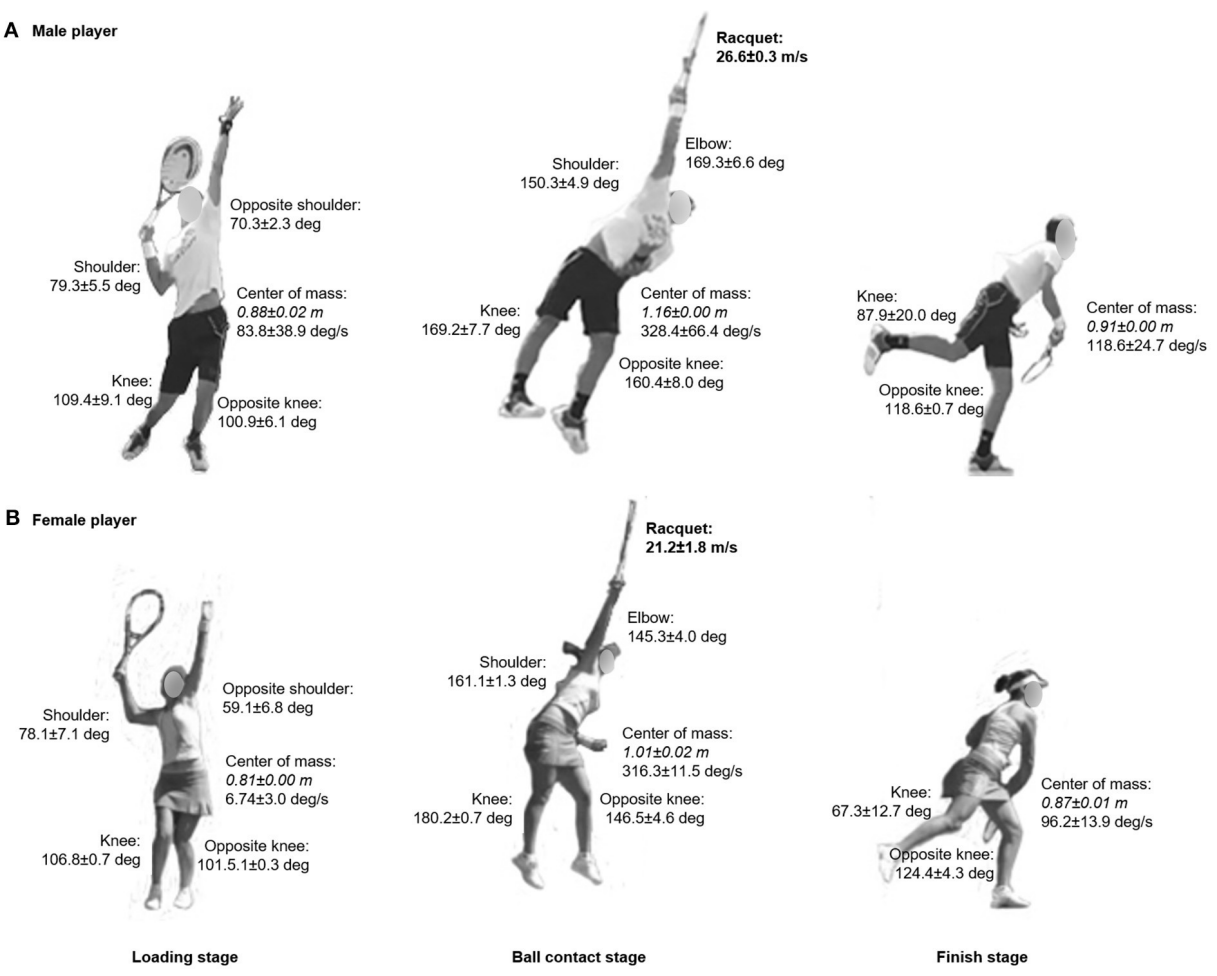
Joint angles, center of mass displacement (in italic) and rotation during loading, ball contact and finish stages of the serve for the male [upper panel, **(A)**] and female tennis players [lower panel, **(B)**]. Racquet velocity (in bold) is also displayed at ball contact stage. Values are means ± SD from trials 1 and 2.

For both female and male tennis players, joint angles, center of mass displacement and rotation during loading, ball contact and finish stages are shown in Figure 1. Racquet velocity at ball contact corresponded to 48.8 ± 1.0 m/s and 46.0 ± 0.9 m/s for the male and female tennis players, respectively.

**Figure 2:**
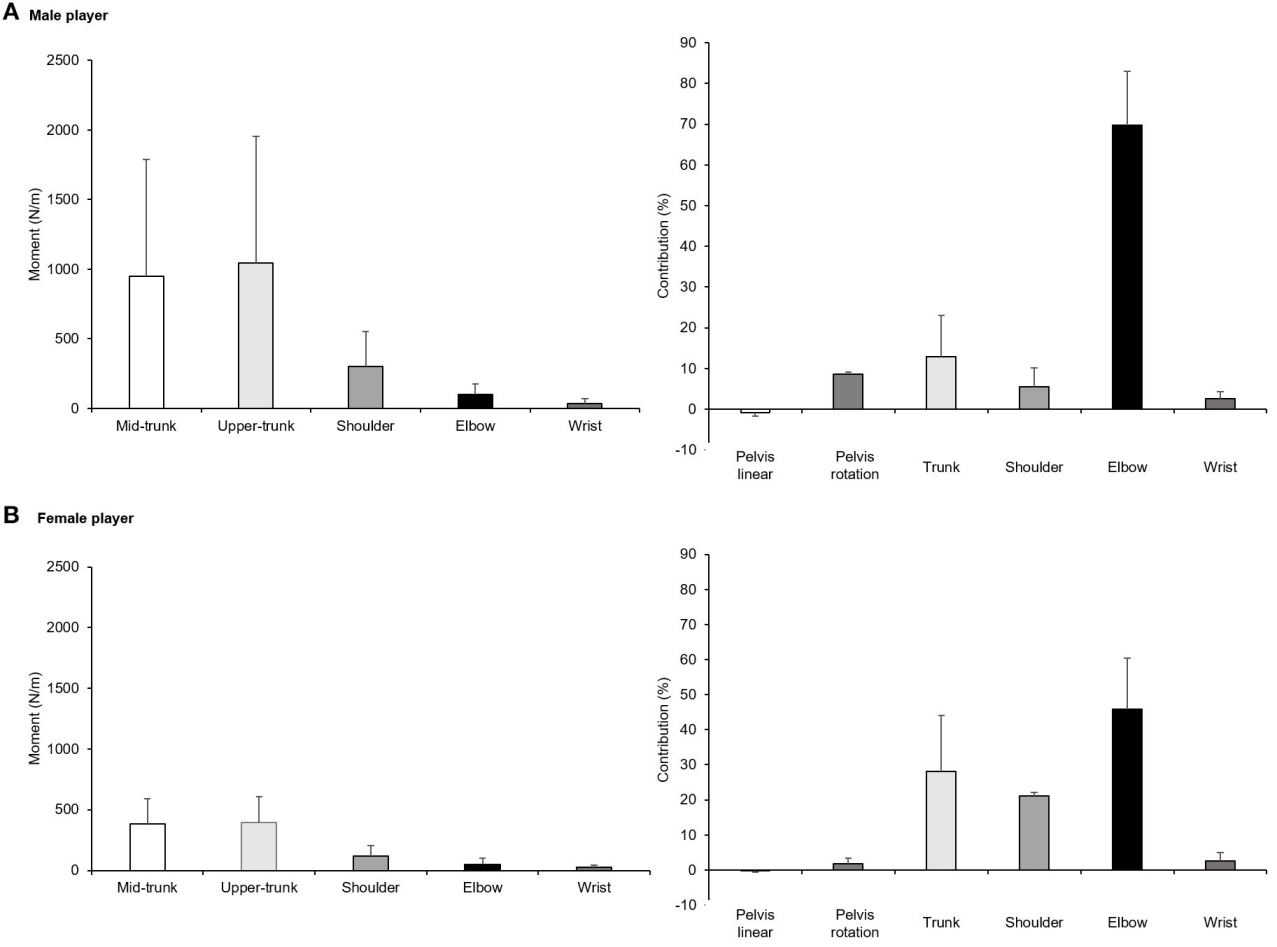
Segmental contributions during loading, ball contact and finish stages of the serve for the male [upper panel, **(A)**] and female tennis player [lower panel, **(B)**]. Values are means ± SD from trials 1 and 2.

Information on the repartition of the articular moments and segmental contributions are included in Figure 2. With the exception of the center of mass rotation at the loading stage, all joint (shoulder, elbow, knee) angles, center of mass displacements and rotations followed a similar development for both participants from loading to finish stages. At ball contact stage, articular moments (mid-trunk, upper-trunk, shoulder, elbow, wrist) and segmental contribution (pelvis linear, pelvis rotation, trunk, shoulder, elbow, wrist) repartitions also showed a comparable movement. Noteworthy, the female tennis player displayed a two-fold lower articular moments and segmental contributions than her male counterpart, except for the trunk and shoulder contributions which appeared double.

Table 1 presents the total body and lower-to-upper body energy transfer for both tennis players. From loading to finish stages, total, lower and upper energy contribution were similar for both players, with CVs deemed acceptable between the two trials.

**Table 1:** Total and lower- to upper-body energetic transfer during tennis flat “first” serve stages for the male and female tennis players.

		**Total energy**		**Lower-body energy**		**Upper-body energy**	
		**Trial 1**	**Trial 2**	**Trial 1**	**Trial 2**	**Trial 1**	**Trial 2**
**Male player**							
Loading stage	Value (J)	909.1	787.7	283.1	273.5	626.0	514.3
	Mean ± SD (J)	848.4 ± 85.8		278.3 ± 6.8		570.1 ± 79.0	
	CV	10.12		2.44		13.86	
	% Trial 1-2	−13.35		−3.39		−17.82	
Ball contact stage	Value (J)	947.4	886.6	338.4	280.7	609.0	605.9
	Mean ±SD (J)	917.0 ± 43.0		309.5 ± 40.8		607.5 ± 2.2	
	CV	4.69		13.19		0.36	
	% Trial 1-2	−6.42		−17.06		−0.51	
Finish stage	Value (J)	985.4	744.7	377.3	277.7	608.2	467.0
	Mean ±SD (J)	865.1 ± 170.2		327.5 ± 70.4		537.6 ± 99.8	
	CV	19.68		21.49		18.57	
	% Trial 1-2	−24.43		−26.38		−23.22	
**Female player**							
Loading stage	Value (J)	967.8	797.2	302.5	279.4	663.5	517.9
	Mean ±SD (J)	882.5 ± 120.6		291.9 ± 17.7		590.7 ± 103.0	
	CV	13.67		6.06		17.43	
	% Trial 1-2	−17.63		−8.21		−21.94	
Ball contact stage	Value (J)	971.3	869.7	365.7	297.8	605.6	571.9
	Mean ±SD (J)	920.5 ± 71.8		331.7 ± 48.0		588.8 ± 23.8	
	CV	7.80		14.47		4.05	
	% Trial 1-2	−10.46		−18.57		−5.56	
Finish stage	Value (J)	930.5	775.3	362.3	302.5	568.3	472.8
	Mean ±SD (J)	852.9 ± 109.8		332.4 ± 42.3		520.6 ± 67.5	
	CV	12.87		12.72		12.97	
	% Trial 1-2	−16.68		−16.50		−16.80	

## Discussion

The main purpose of this case study was to report the feasibility of IMUs kinematic and kinetic data collection and analyze in tennis serve. For a quite similar racquet velocity at ball contact, both female and male tennis players displayed similar joint angles, center of mass displacements and rotation, as well as repartition of the articular moments and segmental contributions (with two-fold lower data for the female participant) during the loading, ball contact and finish stages. Total, lower and upper energy contribution were also similar for both players. Although remaining very descriptive, our data reveal acceptable CVs between two trials of five “first” serves for both female and male tennis players. This IMUs device appears suitable for on-court biomechanical data collection and subsequent analysis to provide tennis players and coaches tailored feedbacks.

Because it is the first study reporting tennis serve whole-body mechanics using IMUs, comparison with the tennis serve literature is challenging. Shoulder and elbow joints angles appear in the range of data measured in experienced junior tennis players at serve (6). Similar shoulder external rotation angle has also been reported in professional tennis players with lower values in male than female (26). Elbow moment also aligns with the ones reported in male and female professional tennis players (26). However, it differs from NCAA Division I male tennis players' flat serve reported by Abrams et al. (27), which may be due to methodological differences (IMUs *vs*. camera markerless motion capture system) and variance in ITF category levels [3–4 vs. 2 (equivalent to the United States Tennis Association's National Tennis Rating Program)]. Hip velocity appears lower than for elite female tennis players (28). Finally, racquet velocity at ball contact appears similar to the one reported in ITF category level 2 and professional male and female players (28, 29). Consequently, wireless and lightweight IMUs showed reasonable similarities compared to the aforementioned studies that used different motion capture systems. Given the existing correlation between kinematic or temporal pattern, (upper) joint kinetics and ball velocity (30) with possible overcompensation exposing to a greater risk of injury (11), IMUs represent promising alternative for ecological on-court 3D measurement in tennis. Additional large-scale validation is warranted to confirm this assumption.

A novel finding with the IMUs is the total and lower-to-upper body energetic transfer during tennis flat “first” serves. It is interesting to note the similar transfer pattern between the male and female tennis players monitored. As observed within the lower-to-upper body energetic transfer, the “*leg drive*” contribution plays a major role in the serve's “*kinetic chain*” (8, 26), influencing the transfer of linear and angular momentum to the trunk, the arm and the racquet (9). Of note, an ineffective transfer might improve the risk of joint injury, especially in the last parts (i.e., shoulder, elbow and wrist) of the “*kinetic chain*” (31). Overall, this indicates that IMUs may be effective to properly collect on-court tennis serve's energy transfer (of note, players tested in this case study did not reported any obstruction/restriction in their movement). Such approach may help to decipher the critical mechanical contributors to tennis serve and offers tennis practitioners individualized biomechanical and technical instructional direction.

For all data collected, the CVs ranged from “*acceptable*” to “*excellent*”. Of course, in such sport-specific technical movement, variability and inter-joint coordination are paramount for a correct execution (32). Mainly governed by the ball toss, this leads to a “funnel-like” control pattern of the critical end-point parameter (33). Accordingly, CVs decreased from lower- to upper-body parts. The regulation of end-point parameters depends on compensatory joint mechanics (28) coupled with perception (i.e., external information) of the action (28). Because variability is only functional within a defined “*window*” (34), tennis serve training should be repeated in varied environment (e.g., pace, spin, direction, and height of ball feed or drill structure) in order to develop motor control processes toward identifying and adopting an optimal serve (35). In this view, IMUs feedbacks might provide essential information in the motor learning process.

From a practical viewpoint, although revealing suitable information to the practitioners, this case study remains mainly descriptive with limited generalizability. Indeed, only comparing tennis serve with IMUs in reference to “*gold standard*” biomechanical systems within larger sample size will clarify the question of validity. Nevertheless, the development of such non-invasive, portable IMUs with semi- or fully-automated modeling algorithm (including outlier detection) for quantifying tennis serve (36), as well as hitting stroke (37, 38) opens new research avenues in term of load monitoring and management in tennis that are crucial for limiting injury risk.

To summarize, this case study indicates the potential usefulness of IMUs as analytic tool in tennis serve. With acceptable coefficient of variation between two trials of five flat “first” serves, ascertaining the major biomechanical variables of on-court tennis serve with IMUs may aid practitioners to facilitate motor learning process and develop serve efficiency through more biomechanically driven remediation and feedback.

## Data availability statement

The raw data supporting the conclusions of this article will be made available by the authors, without undue reservation.

## Ethics statement

The studies involving human participants were reviewed and approved by Local Ethical Committee. Written informed consent to participate in this study was provided by the participants' legal guardian/next of kin.

## Author contributions

Conceptualization, methodology, software, and investigation: DD. Writing—original draft preparation: FB. Writing—review and editing: FB and DD. Both authors have read and agreed to the published version of the manuscript.

## Conflict of interest

The authors declare that the research was conducted in the absence of any commercial or financial relationships that could be construed as a potential conflict of interest.

## Publisher's note

All claims expressed in this article are solely those of the authors and do not necessarily represent those of their affiliated organizations, or those of the publisher, the editors and the reviewers. Any product that may be evaluated in this article, or claim that may be made by its manufacturer, is not guaranteed or endorsed by the publisher.
